# Cash incentives versus defaults for HIV testing: A randomized clinical trial

**DOI:** 10.1371/journal.pone.0199833

**Published:** 2018-07-06

**Authors:** Juan Carlos C. Montoy, William H. Dow, Beth C. Kaplan

**Affiliations:** 1 Department of Emergency Medicine, University of California, San Francisco, San Francisco, California, United States of America; 2 Division of Health Policy and Management, School of Public Health University of California, Berkeley, Berkeley, California, United States of America; The Ohio State University, UNITED STATES

## Abstract

**Background:**

Tools from behavioral economics have been shown to improve health-related behaviors, but the relative efficacy and additive effects of different types of interventions are not well established. We tested the influence of small cash incentives, defaults, and both in combination on increasing patient HIV test acceptance.

**Methods and findings:**

We conducted a randomized clinical trial among patients aged 13–64 receiving care in an urban emergency department. Patients were cross-randomized to $0, $1, $5, and $10 incentives, and to opt-in, active-choice, and opt-out test defaults. The primary outcome was the proportion of patients who accepted an HIV test. 4,831 of 8,715 patients accepted an HIV test (55.4%). Those offered no monetary incentive accepted 51.6% of test offers. The $1 treatment did not increase test acceptance (increase 1%; 95% confidence interval [CI] -2.0 to 3.9); the $5 and $10 treatments increased test acceptance rates by 10.5 and 15 percentage points, respectively (95% CI 7.5 to 13.4 and 11.8 to 18.1). Compared to opt-in testing, active-choice testing increased test acceptance by 11.5% (95% CI 9.0 to 14.0), and opt-out testing increased acceptance by 23.9 percentage points (95% CI 21.4 to 26.4).

**Conclusions:**

Small incentives and defaults can both increase patient HIV test acceptance, though when used in combination their effects were less than additive. These tools from behavioral economics should be considered by clinicians and policymakers. How patient groups respond to monetary incentives and/or defaults deserves further investigation for this and other health behaviors.

## Registration

Clinical Trials NCT01377857.

## Introduction

Behavioral economics approaches such as defaults and incentives for changing patient behavior have been implemented across a wide range of clinical settings. Monetary incentives have been employed as a means to modify health-related behaviors in substance abuse treatment [1], smoking cessation [2], weight loss [3], risky sexual behavior [4, 5], and some one-time or infrequent behaviors such as immunization [6] and HIV screening [7]. Defaults have likewise been shown to be effective at influencing behaviors; for example, the prescribing of generics over brand-name medications [8, 9], end-of-life decisions in advance directives [10], and participation in diabetes care [11]. Because both incentives and defaults have proven effective, further research is needed to develop our understanding regarding which types of interventions are more effective at changing specific types of behaviors–a question best answered through at-scale head-to-head experimentation [12].

This paper analyzes a head-head randomized trial of approaches to increase HIV testing among emergency department patients. Identifying HIV infections remains a top priority in addressing the ongoing HIV epidemic [13–15], but despite widespread agreement that universal opt-out screening should be adopted [16–19], failure to screen is the norm across all hospital types [20, 21]. A previous publication using a subset of data from this trial (arms with no monetary incentives) found that changing defaults for HIV testing yielded clinically significant differences in HIV testing [22]. Here we estimate the extent to which various cash incentives increase HIV test acceptance, compare this effect head-to-head with the effect of defaults, and analyze whether incentives and defaults can be used together to optimize test acceptance.

## Methods

We conducted a randomized clinical trial in the emergency department of an urban teaching hospital and regional trauma center. Between June 18, 2011, and June 30, 2013, non-clinical staff approached patients in the emergency department: once to offer a rapid HIV test and once for a questionnaire. Patients were identified and approached by study staff during times not interfering with their clinical care. Accepted tests were completed as part of their care in the department. The ten-minute self-administered questionnaires were described generically as improving emergency department care. After both the test and questionnaire responses were recorded, patients were fully debriefed and written consent was obtained. Per state and federal law, and with the approval of the institutional review board (IRB), minors were able to consent to the study. The study received IRB approval from the University of California, San Francisco, was conducted and reported in accordance with CONSORT guidelines, and was registered as clinicaltrials.gov study NCT01377857. The protocol has been described previously and presented in greater detail in S1 Text [22].

Monetary incentives were assigned at the zone-day level: all patients in each of the four ED zones on a given day received the same treatment assignment. Incentives were assigned to each zone using a random-number generator, independent from the other zone assignments.

A random number generator was used to create default wording (opt-in, active-choice, and opt-out) treatment assignments, randomized at the patient level, each with equal probability. Patients were also randomly assigned to be offered the questionnaire either before or after the HIV test offer. No incentive was offered for questionnaire completion. The incentive, default, and questionnaire timing treatment assignments were cross-randomized in a factorial design.

Study staff began each shift in one of four emergency department zones and approached all eligible patients in that zone prior to moving to the next zone. The starting zone was determined at the day level using a random-number generator, in which each zone had a 25% chance of being the starting zone any given day. Staff were not blinded to treatment assignments.

### Participants

Study inclusion criteria were: age 13–64, able to consent to HIV testing and study inclusion, and English- or Spanish-speaking. Patients were excluded if known HIV-positive, had tested for HIV in past three months, pregnant, in police custody, or had participated in this study in the previous three months.

### Protocol

Using a standardized script, study staff informed patients that the emergency department was offering rapid screening HIV tests. Patients were told that the testing was non-targeted and routine, and used a rapid assay with results available during their ED visit, approximately 1–2 hours. The test offer followed: opt-in “You can let me, your nurse, or your doctor know if you'd like a test today,” active-choice “Would you like a test today?” or opt-out “You will be tested unless you decline.” Finally, if the patient was assigned to a positive monetary incentive, they were informed, “To encourage testing today we are offering a $1 cash incentive” (substituting $5 or $10 as relevant). No mention of monetary incentives was made to patients who were assigned to the $0 treatment.

Study staff notified clinicians of patients accepting HIV tests. No pre-test counseling was performed. Patients were informed of negative test results by their nurse or clinician. Positive test results were disclosed by the patient’s clinician in accordance with the protocol established by the hospital's HIV Rapid Testing and Referral Program.

### Statistics

The primary outcome was test acceptance percentage. Treatment effects were estimated with univariate and multivariable ordinary least squares regression. Tables report raw linear regression coefficients, which are directly interpretable as the difference in the proportion of subjects who accept an HIV test; interaction effects are similarly straightforward to interpret [23].

We also examined effects across HIV risk subgroups, per approximated Denver HIV Risk Score (S1 Table) [24, 25]. Scores depend on demographics (age, gender, race/ethnicity), risk behaviors (sex with a male, vaginal intercourse, receptive anal intercourse, IV drug use), and past HIV testing. We classified patients as low risk (score under 20), intermediate risk (scores 20–39), and high risk (scores 40 or higher). For patients who did not complete the questionnaire, the risk score was estimated using available data only. While analysis by risk level was a planned analysis, the Denver HIV Risk Score was published and validated during our data collection, so these risk definitions were not pre-specified. Because patient responses within the same zone and on the same day could be correlated, we clustered standard errors by day and emergency department zone (zone-day level). Sensitivity analyses, including different model specifications using ordinary least squares and multivariable logistic regression, are presented in the Supporting Information. Randomization and all analyses were performed using STATA 13.1.

Planned sample size was sufficient to detect a 5 percentage point difference in test acceptance between treatment arms with 80% power at a 5% significance level between the no incentive treatment assignment and one of the positively-valued incentive assignments within one of the default assignments. This 5 percentage point effect size was the minimum difference we deemed to be clinically important. We assumed a baseline test acceptance percentage of 50%. This predicted a sample size of 2,349 for the no incentive group and 1,175 for each of the incentive groups (no incentive was designed to have a greater quantity than each of the positively-valued incentive arms). These sample sizes yield a total of 5,874 patients within each default group, for a total of 17,622 patients in the study. Our actual enrolled sample size was smaller than originally planned due to enrollment difficulties.

## Results

### Participation and randomization

**Fig 1:**
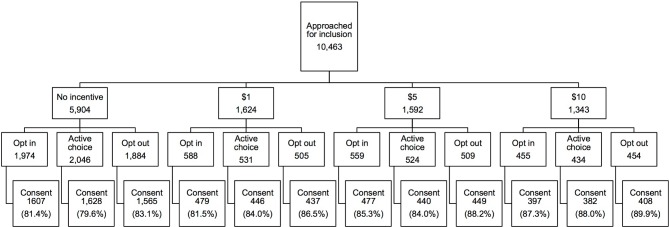
Flow diagram. Of 10,463 patients approached for inclusion in study, 8,715 consented. Because patients were retrospectively consented, no patients were excluded after being consented for inclusion.

Research assistants approached 10,463 patients to offer HIV tests and questionnaires. 8,715 (82.3%) of patients consented to inclusion in the study. Randomization yielded no significant differences in demographic groups across monetary incentive treatment assignments (Table 1); demographics according to default assignment are presented in S2 Table. The distributions of demographics and chief complaints did not vary by assignment to monetary incentive. Fig 1 shows the flow of patients through treatment assignments, with consent rates for each incentive-default combination.

**Table 1:** Demographics.

VARIABLES	(1) All subjects	(2) No incentive	(3) $1	(4) $5	(5) $10
Male	5192 (59.6)	2887 (60.1)	811 (59.5)	798 (58.4)	696 (58.6)
Age	40 (30–52)	32 (40–53)	41 (30–53)	41 (29–51)	42 (29–52)
American Indian / Alaska Native	105 (1.2)	59 (1.2)	14 (1.0)	15 (1.1)	17 (1.4)
Asian	817 (9.4)	451 (9.4)	132 (9.7)	130 (9.5)	104 (8.8)
Black	2256 (25.9)	1249 (26.0)	346 (25.4)	341 (25.0)	320 (27.0)
Native Hawaiian / Pacific Islander	259 (3.0)	140 (2.9)	33 (2.4)	41 (3.0)	45 (3.8)
White	4894 (56.2)	2676 (55.7)	785 (57.6)	777 (56.9)	656 (55.3)
Unreported	585 (6.7)	330 (6.9)	84 (6.2)	90 (6.6)	81 (6.8)
Latino^a^	2152 (24.7)	1163 (24.2)	338 (24.9)	355 (26.0)	295 (24.9)
Spanish	1048 (12.0)	572 (11.9)	176 (12.9)	170 (12.4)	130 (11.0)
High school completion	5256 (60.3)	2844 (59.3)	846 (62.1)	827 (60.5)	739 (62.3)
LGBT	1028 (11.8)	589 (12.3)	164 (12.0)	149 (10.9)	126 (10.6)
Chief complaint Abdominal	1775 (20.4)	979 (20.4)	296 (21.7)	265 (19.4)	235 (19.8)
Cardiovascular	1020 (11.7)	544 (11.3)	176 (12.9)	167 (12.2)	133 (11.2)
Endocrine	107 (1.2)	61 (1.3)	16 (1.2)	12 (0.9)	18 (1.5)
General / other	572 (6.6)	288 (6.0)	88 (6.5)	106 (7.8)	87 (7.3)
GU / renal	509 (5.8)	302 (6.3)	69 (5.1)	71 (5.2)	67 (5.6)
Musculoskeletal	1388 (15.9)	763 (15.9)	210 (15.4)	212 (15.5)	203 (17.1)
Stroke	30 (0.3)	18 (0.4)	2 (0.1)	5 (0.4)	5 (0.4)
Neurologic non-stroke	523 (6.0)	296 (6.2)	64 (4.7)	82 (6.0)	81 (6.8)
Oral / dental	129 (1.5)	69 (1.4)	21 (1.5)	17 (1.2)	22 (1.9)
Psychiatric	87 (1.0)	52 (1.1)	13 (1.0)	12 (0.9)	10 (0.8)
Respiratory	660 (7.6)	372 (7.8)	111 (8.1)	94 (6.9)	83 (7.0)
Skin	651 (7.5)	386 (8.0)	76 (5.6)	106 (7.8)	83 (7.0)
Substance use	196 (2.2)	92 (1.9)	46 (3.4)	33 (2.4)	25 (2.1)
Trauma	799 (9.2)	422 (8.8)	132 (9.7)	140 (10.2)	105 (8.8)
Did not complete questionnaire	1689 (19.4)	940 (19.6)	268 (19.7)	238 (17.4)	243 (20.5)
Risk Category Low	3510 (40.3)	1943 (40.5)	537 (39.4)	554 (40.6)	576 (48.5)
Intermediate	4394 (50.4)	2388 (49.8)	695 (51.0)	697 (51.0)	614 (51.7)
High	811 (9.3)	469 (9.8)	130 (9.5)	115 (8.4)	97 (8.2)
Previously tested for HIV	7049 (80.9)	3880 (80.8)	1105 (81.1)	1114 (81.6)	950 (80.0)
Observations	8,715	4,800	1,362	1,366	1,187

Each cell contains number (percentage); age is median and (25–75% interquartile range)

LGBT = self-identified lesbian, gay, bisexual, or transgender

Race adds to greater than 100% because each respondent could report multiple races.

^a^ Latino is categorized as an ethnicity, separate from race.

### Treatment effects

**Fig 2:**
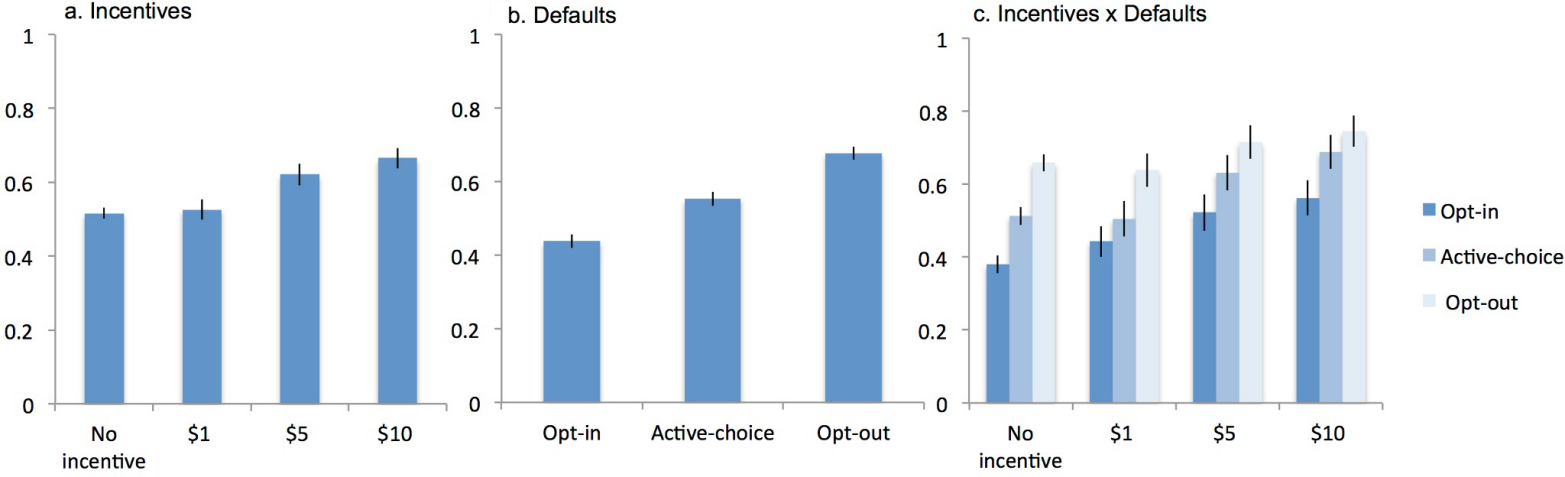
HIV consent by treatment assignment. Proportion of patients accepting an HIV test according to treatment assignment: 2a monetary incentives, 2b defaults, and 2c incentive x default combinations.

HIV tests were accepted by 4,831 patients (55.4%). Those offered no monetary incentive accepted 51.6% of test offers; those offered $1, $5, and $10 accepted 52.6%, 62.1%, and 66.6% of tests, respectively. These unadjusted differences showin in Table 2, Column 1 and Fig 2 reflect an absolute difference between the $1 treatment and no incentive treatment of 1% (95% confidence interval -2.0 to 3.9); the $5 and $10 treatments increased test acceptance rates by 10.5 and 15 percentage points, respectively (95% CI 7.5 to 13.4 and 11.8 to 18.1). Patients in the opt-in scheme accepted 43.8% of test offers, unadjusted for incentives. Patients in the active-choice scheme were 11.5 percentage points more likely to accept test offers (95% CI 9.0 to 14.0); those in the opt-out scheme were 23.9 percentage points more likely to accept testing (95% CI 21.4 to 26.4).

**Table 2:** OLS raw differences.

Variables	Incentives (1)	Defaults (2)	Incentives and defaults (3)	Incentives, defaults, and interactions (4)
Incentives				
$1	0.01 (0.016)		0.012 (0.016)	0.062** (0.025)
$5	0.105*** (0.017)		0.106*** (0.016)	0.142*** (0.029)
$10	0.150*** (0.016)		0.147*** (0.016)	0.182*** (0.027)
Defaults				
Active-choice		0.115*** (0.013)	0.117*** (0.013)	0.133*** (0.018)
Opt-out		0.239*** (0.013)	0.239*** (0.013)	0.279*** (0.017)
Incentives x Defaults				
$1 x Active-choice				-0.071** (0.035)
$1 x Opt-out				-0.083** (0.036)
$5 x Active-choice				-0.023 (0.037)
$5 x Opt-out				-0.086** (0.040)
$10 x Active-choice				-0.006 (0.038)
$10 x Opt-out				-0.095*** (0.036)
Constant	0.516*** (0.008)	0.437*** (0.010)	0.399*** (0.011)	0.380*** (0.013)
Observations	8,715	8,715	8,715	8,715

Dependent variable = acceptance of HIV test. Each column shows percentage point difference in HIV test acceptance estimated from an ordinary least squares regression (standard error). Omitted categories for incentives, defaults, and risk groups: no incentive, opt-in, and low risk, respectively.

Standard errors are clustered at zone-day level.

*** p<0.01,

** p<0.05,

* p<0.1

Incentives and defaults are considered jointly under a model without interaction terms and a model with them (Table 2, Columns 3 and 4, respectively). The estimates of the effects of monetary incentives and of defaults are similar in the multivariable model without interactions (Table 2, Column 3) to the estimates from each of the univariate models. When the effects of incentives are measured separately for each default (Table 2, Column 4), each of the cash incentives have the largest effect within the opt-in group. The $1 incentive was associated with a 6.2 percentage point increase in test acceptance (95% CI 1.4 to 11.0); it did not increase test acceptance among the active-choice or the opt-in group. The effects of the $5 and $10 incentives were attenuated in the opt-out group.

### Risk of infection

The sample of patients enrolled in the study was comprised of 40.3% low-risk, 50.4% intermediate-risk, and 9.3% high-risk patients. Univariate analysis shows that intermediate-risk patients were 7.1, and high risk were 9.1 percentage points more likely to test than low-risk patients (95% CI 5.0 to 9.3 and 5.3 to 12.8, respectively).

When the effect of incentives is calculated separately for each group, the estimates show a similar pattern to the results from the univariate model: the $1 incentive has no effect on testing, and the $5 and $10 each increase test acceptance. None of the interaction terms is significantly different from 0, suggesting that the monetary incentives affected behavior equally across risk groups. Sensitivity analyses are presented in the supplementary material: risk-specific interaction terms (S3 Table), estimation with a logistic regression (S4 Table), and a back-of-the-envelope calculations to account for differential study participation rates (S5 and S6 Tables).

**Fig 3:**
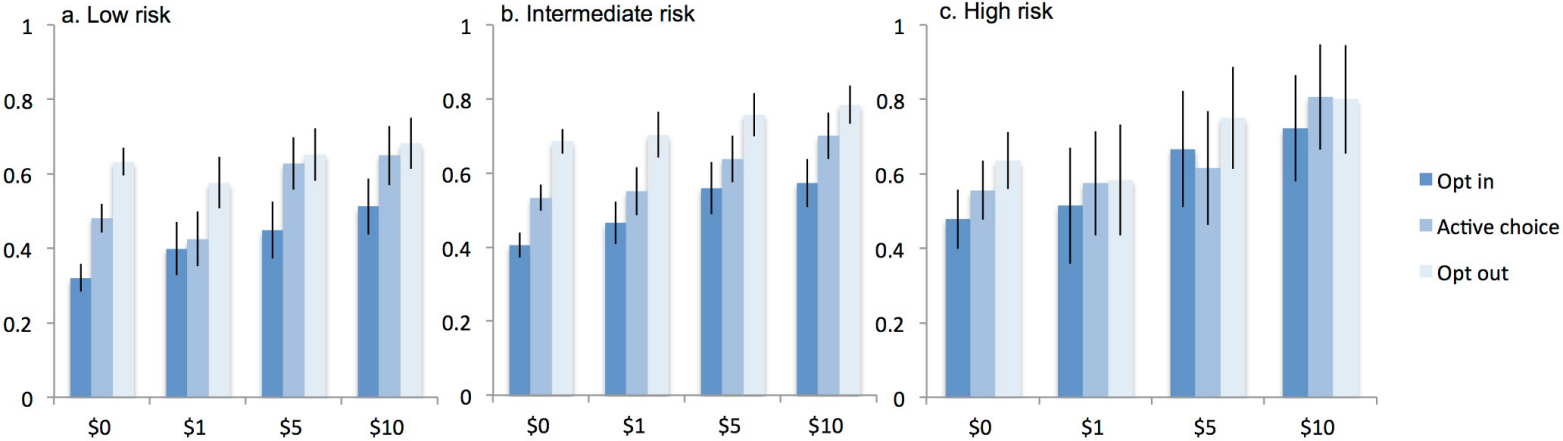
HIV consent by incentive-default treatment assignment and risk of infection. Proportion of patients accepting an HIV test according to incentive-default treatment assignment, stratified by risk group. Risk of infection was estimated by the Denver HIV Risk Score: < 20 low risk, 20–39 intermediate risk, ≥40 high risk.

Fig 3 presents results from a model that estimates the effects of incentives on test uptake separately for each default within patients from each risk category: coefficients were estimated for incentives, defaults, and risk level, and each two-way and three-way interaction between them.

## Discussion

This study tested two types of behavioral economics interventions–monetary incentives and defaults–and found evidence that each can be effective in increasing HIV test uptake. This is to our knowledge the first study to directly compare two types of behavioral economics interventions in any health behavior context. Recent research has evaluated how to target a single type of intervention, but has not yet compared different types of interventions [26]. In large part this literature has explored repeated behaviors or behavior over time, such as medication adherence and weight loss [27, 28].

The interventions were tested both separately and in combination with each other with a rigorous design that included random assignment to small monetary incentives and patient-level randomization to a one-sentence variation in test offer, with all else held constant. The effects were persistent across all model specifications and levels of patient risk of infection, though the effects were somewhat attenuated when defaults and incentives were used together: the $1 incentive increased test acceptance in the opt-in but not the other default settings, and the $5 and $10 incentives were less effective under the opt-out default than the other default settings. In general, higher-risk patients tested at higher rates than lower-risk patients and had smaller responses to treatments. Among all treatment assignments, opt-out had the largest effect, followed by the $10 incentive.

Compared to previously published work from this study, which demonstrated that defaults significantly affect patient behavior, this study places two classes of behavioral economics nudges in direct comparison with nearly double the sample size. We again confirmed that active-choice is a category distinct from opt-in, both providing policymakers with clearer guidance on how to implement policies and also bringing this field in closer alignment with the existing literature in psychology and economics [29, 30]. Despite being universally present in health care, defaults have been understudied in medicine and this topic deserves further attention.

The proportion of patients accepting testing may vary in other settings and with other populations as compared to those within this single-center study. However, patients with a wide range of demographics, chief complaints, and risk factors for HIV were included in the study. Although the particular percentages may be quite different, the patterned responses to small monetary incentives and also to op-in, active-choice, and opt-out test offers may be expected for HIV testing in other settings as well as for decisions about other medical tests.

By blinding patients to the study itself and also to its components, the retrospective informed consent design has the advantage of minimizing or even eliminating many potential sources of bias but introduces the risk of bias from post-randomization withdrawal. We see evidence of this: the proportion of approached patients who participated in the study increases monotonically with monetary incentives. However, the difference in participation rates is small and did not drive the results here; sensitivity analysis did not change the primary results.

The three monetary incentive values used in this study are somewhat arbitrary but are on a scale that might reasonably be chosen by a hospital or health system. The $1 serves to test if, as previously found [7], whether a monetary incentive is offered is more important than the value of the incentive–a finding we did not replicate. We chose immediate cash incentives in order to maximize the response under the prediction from behavioral economics that equivalent payments such as a check given immediately or cash given later would likely yield smaller increases in test acceptance rates, as would a deduction of the same dollar amount from one’s hospital bill.

Our ED population had few barriers to testing: there was no travel time, scheduling, written consent, or, in most cases, additional blood draws. But, even under the $10, opt-out treatment assignment, test uptake did not approach 100%. This result is cause for pessimism about the potential for small incentives, defaults, or both to achieve the target of universal screening. This suggests that some patients truly believe the test is not worthwhile, and for others the psychological costs of learning one’s HIV status are too high. This poses a challenging question of how to achieve universal testing and identify all existing cases of HIV infection. Nevertheless, among high-risk patients the combination of incentives and defaults raised test acceptance from 48% in the $0 opt-in arm to 80% in the $10 opt-out arm.

This study directly compares two behavioral economics interventions and adds to the existing evidence that small interventions can have significant effects in directing patients toward more optimal health-related behaviors. Our results have the potential to help inform how to structure HIV test offers for other emergency departments as well as other health care settings. The finding that, on average, moving from opt-in to opt-out testing influenced behavior more than even the largest incentive reinforces the notions that the medicine is not just a transaction, and what we say to patients matters. This field is still relatively new, and much remains to be learned about how and in what settings to use behavioral economics approaches to improving health-related behavior. How patients respond to monetary incentives and defaults deserves further investigation for this and other health problems.

## Supporting information

S1 Text Protocol. (DOCX)

S1 Table Denver risk score components. (DOCX)

S2 Table Demographics. (DOCX)

S3 Table Risk-specific results. (DOCX)

S4 Table Alternate models. (DOCX)

S5 Table Sensitivity analysis. (DOCX)

S6 Table Sensitivity analysis. (DOCX)
