# Supplementary material for: Cash incentives versus defaults for HIV testing: A randomized clinical trial
Source: PLoS One. 2018 Jul 6;13(7):e0199833. doi: 10.1371/journal.pone.0199833 (PMC6034801; doi:10.1371/journal.pone.0199833)
Supplement: S1 Text — (DOCX) [file pone.0199833.s001.docx]

**S1 Text. Study protocol**

**A. Overview**

Patients were engaged twice over the course of their time in the emergency department (ED) in parallel with usual ED care: once to offer them a questionnaire and once to offer them a rapid HIV test. Eligible patients were assigned to a treatment triad: an offer of an HIV test with one of three scripts (*Defaults*), an *Incentive*value, and the timing of a *Questionnaire*.   Each of these sets of treatments was cross-randomized with the others in a factorial design. Patients were blinded to the study until after both the HIV test and questionnaire were offered; they were then debriefed and asked to provide informed consent. The study received institutional review board approval from the University of California, San Francisco (which the University of California, Berkeley IRB relied on), and was registered as clinicaltrials.gov study NCT01377857.

**B. Patient Selection**

Research assistants used the electronic health record to identify patients who satisfied the selection criteria. Patients were eligible for inclusion in the study if they were between 13 and 64 years old, able to consent to HIV testing, and spoke either English or Spanish. Patients were excluded if they had been previously diagnosed with HIV infection, had received an HIV test in the previous three months, were pregnant, were in police custody, or participated in this study in the previous three months. For patients presenting with altered mental status, the ability to consent was determined by the study staff with the patient’s nurse or clinician at the time of approach. Study staff approached prospective patients according to patient availability (e.g., not undergoing procedure or away at radiology). Staff started each shift in one of four emergency department zones (Zones 1-4) according to a calendar created using a random-number generator to assign starting zones with equal probability. After exhausting all eligible patients in a given zone, staff moved to the next higher-numbered zone (or from Zone 4 to 1). Three non-overlapping five-hour shifts were scheduled each day with staff working 20 shifts per week.

**C. Intervention**The test offer was initiated with the same script for every patient: “We’re offering routine HIV tests to all of our patients. It’s a rapid test with results available in 1-2 hours.” This was followed by the test offer according to the *Default* assignment, and subsequent monetary incentive (if greater than $0).

**C.1. Defaults**.  The HIV test was offered according to one of three scripts: *opt-in*, *active choice*, or *opt-out*.

*Opt-in*: default of no test – patients must ask for the test in order to receive it. Patients were informed of the availability of rapid testing.  They were tested only if they request the test.

“You can let me, your nurse, or your doctor know if you'd like a test today.”

Active-choice: no default – patients must actively accept or actively decline the test.

“Would you like a test today?”

Opt-out: default to test – patients were informed that they will receive a rapid HIV screening test unless they opt out.

“You will be tested unless you decline.”

Randomization occurred at the patient level. A random number generator was used to create individual assignments with equal probability. These assignments were listed on a log sheet for each shift, to which patients were added sequentially.

**C.2. Monetary incentives**.  Monetary incentives were assigned at the day-zone level using a random number generator under a cluster-randomization scheme with each zone as a cluster: each of the four zones of the emergency department had an incentive value assigned to it each day. Assignments were weighted for the ratio 2:1:1:1 for the $0, $1, $5, and $10 assignments. Patients in a given zone were only informed of the monetary incentive that applied to them, and patients in the no-incentive treatment were not informed of the possibility of monetary incentives.

Patients in zones assigned to cash incentives were informed immediately after the HIV test offer that the ED was offering cash incentives to promote HIV testing, and informed of the value.   Patients who agreed to get tested received this cash incentive; those who declined testing did not.  Incentives were disbursed by study staff as cash. Payments were denominated as $1, $5, or $10.  There was no cash incentive for participation in the study.

**C.3. Questionnaire.**Separately from the test offer, patients were approached by a member of the research team and asked to complete a short (5-10 minutes) questionnaire. The questionnaires were administered at one of two timing treatments – a) before the patient was offered an HIV test (*Early questionnaire*) or b) after the patient was offered an HIV test (*Late questionnaire*). This timing assignment was cross-randomized with all other treatments.  Patients were informed that the questionnaire was confidential but not anonymous, that it was not part of their medical record, and that their participation answers would in no way influence the care they received.

**D. HIV Test**

Tests were performed by the SFGH Clinical Microbiology Laboratory, which processes all blood work for the emergency department with the exception of less common send-out labs. The Laboratory used the Uni-Gold Recombigen HIV test, with a sensitivity of 100% (99.5-100) and a specificity of 99.7% (99.0-100), according to the product insert.  This is considered a diagnostic test by the FDA, but all patients with a positive result will have further, confirmatory testing.

**D.1. Test results.**  Patients were notified of their test results during their visit to the ED. The patient’s nurse or clinician notified them of negative results. Positive disclosures followed the protocol established by the HIV Rapid Testing and Referral Program for SFGH: physicians notified patients that their test resulted in a preliminary positive, and that further testing was required before a definitive diagnosis could be made.  Disclosures were performed by the patient’s physician with the help of members of the Positive Health Access to Services and Treatment (PHAST) team weekdays 8am – 5pm and with the help of Social Work during nights and weekends.  The PHAST team arranged a follow-up appointment at the hospital’s HIV clinic.

**E. Electronic Health Data**

The University of California, San Francisco’s The Health Record Electronic Data Service (THREDS) was employed to securely query data for periods prior to (one year) and after (6 months) the study period for all patients presenting to the emergency department in order to determine baseline HIV-testing trends.

**F. Consent**

Patients were asked to consent to the study retrospectively, while still in the ED.   After the questionnaire and HIV test were offered, a full debriefing and post-study fully-informed written consent was obtained, irrespective of HIV test acceptance, questionnaire timing, and questionnaire participation.  Consent for an HIV test was not a part of consenting for this study; for example, patients could take part in the study and decline the test, or could get tested without taking part in the study.  As per SFGH policy and consistent with state law, patients verbally consented to the HIV test.  Patients received a consent form that explained the full extent of the study: questionnaire and test offer, including defaults and incentives.  Patients who wished to retract their data from the study sample were allowed to do so.  It was stressed to patients that study participation would in no way influence the medical care they receive while in the ED.
