# Supplementary material for: Cash incentives versus defaults for HIV testing: A randomized clinical trial
Source: PLoS One. 2018 Jul 6;13(7):e0199833. doi: 10.1371/journal.pone.0199833 (PMC6034801; doi:10.1371/journal.pone.0199833)
Supplement: S1 Table — (DOCX) [file pone.0199833.s002.docx]

**S1 Table. Denver HIV Risk Score components**

The Denver HIV Risk Score was estimated for each patient using information from the electronic medical record (EMR) and questionnaire responses. For patients who did not complete the questionnaire, the Risk Score was estimated using only data available in the EMR.

| **Table S1.** Denver Risk Score | |
| --- | --- |
| **Variable** | Points |
| Age |  |
| <22 or >60 | 0 |
| 22-25 or 55-60 | 4 |
| 26-32 or 47-54 | 10 |
| 33-46 | 12 |
| Gender |  |
| Male | 21 |
| Race / Ethnicity |  |
| Black | 9 |
| Hispanic | 3 |
| Other | 0 |
| White | 0 |
| Sexual Practices |  |
| Sex with a male | 22 |
| Vaginal intercourse | -10 |
| Receptive anal intercourse | 8 |
| Other risk factors |  |
| Injection drug use | 9 |
| Past HIV testing | -4 |
|  |  |
