# Supplementary material for: Cash incentives versus defaults for HIV testing: A randomized clinical trial
Source: PLoS One. 2018 Jul 6;13(7):e0199833. doi: 10.1371/journal.pone.0199833 (PMC6034801; doi:10.1371/journal.pone.0199833)
Supplement: S2 Table — (DOCX) [file pone.0199833.s003.docx]

**S2 Table. Demographics**

This study used a non-standard consent method: patients were randomized and underwent study procedures prior to consent. This design was chosen to minimize selection bias, reduce the likelihood of directly influencing test acceptance behavior, and minimize Hawthorne effects. While this design more accurately replicates what clinicians might do in practice (i.e., offer an HIV test without providing any prior intervention or mention of a study), this raises the possibility of post-randomization withdrawal. Supplemental Table S1 presents the demographics of enrolled patients according to each set of treatment assignments. We tested each set of treatment groups for systematic differences that could account for different HIV test acceptance. No tests of differences across the incentives groups are significant at the 0.05 level. Race is significant at the 0.05 level for the default treatments; this is largely driven by the unknown and white categories of race. The p-values reported in the table have not been adjusted for multiple testing.

| **S2 Table. Demographics** | | | |  |  |  |  |  |  |  |  |
| --- | --- | --- | --- | --- | --- | --- | --- | --- | --- | --- | --- |
|  | | **All subjects** | **Incentives** | | | |  | **Defaults** | | |  |
|  | | (1) | (2) | (3) | (4) | (5) |  | (6) | (7) | (8) |  |
| VARIABLES | | All subjects | No incentive | $1 | $5 | $10 | *p* | Opt in | Active choice | Opt out | *p* |
| Male | | 5192 | 2887 | 811 | 798 | 696 | 0.61 | 1784 | 1749 | 1659 | 0.12 |
|  | | 0.596 | 0.601 | 0.595 | 0.584 | 0.586 |  | 0.603 | 0.604 | 0.58 |  |
| Age median | | 40 | 40 | 41 | 41 | 42 | 0.23 | 41 | 41 | 42 | 0.70 |
| (25-75%) | | (30 - 52) | (32 - 53) | (30 - 53) | (29 - 51) | (29 - 52) |  | (29 - 52) | (30 - 53) | (30 - 52) |  |
| American Indian / Alaska Native | | 105 | 59 | 14 | 15 | 17 | 0.89 | 35 | 34 | 36 | <0.001 |
|  | | 0.012 | 0.012 | 0.010 | 0.011 | 0.014 |  | 0.012 | 0.018 | 0.013 |  |
| Asian | | 817 | 451 | 132 | 130 | 104 |  | 241 | 284 | 292 |  |
|  | | 0.0937 | 0.094 | 0.097 | 0.095 | 0.088 |  | 0.081 | 0.098 | 0.102 |  |
| Black | | 2256 | 1249 | 346 | 341 | 320 |  | 761 | 711 | 784 |  |
|  | | 0.259 | 0.260 | 0.254 | 0.250 | 0.270 |  | 0.257 | 0.246 | 0.274 |  |
| Native Hawaiian / Pacific Islander | | 259 | 140 | 33 | 41 | 45 |  | 89 | 71 | 99 |  |
|  | | 0.0297 | 0.029 | 0.024 | 0.030 | 0.038 |  | 0.03 | 0.250 | 0.35 |  |
| White | | 4894 | 2676 | 785 | 777 | 656 |  | 1707 | 1637 | 1550 |  |
|  | | 0.562 | 0.557 | 0.576 | 0.569 | 0.553 |  | 0.577 | 0.265 | 0.542 |  |
| Unreported | | 585 | 330 | 84 | 90 | 81 |  | 176 | 235 | 174 |  |
|  | | 0.0671 | 0.0688 | 0.0617 | 0.0659 | 0.0682 |  | 0.06 | 0.081 | 0.061 |  |
| Latino | | 2152 | 1163 | 338 | 355 | 295 | 0.61 | 756 | 688 | 708 | 0.28 |
|  | | 0.247 | 0.242 | 0.249 | 0.260 | 0.249 |  | 0.255 | 0.238 | 0.248 |  |
| Spanish | | 1048 | 572 | 176 | 170 | 130 | 0.46 | 372 | 348 | 328 | 0.44 |
|  | | 0.120 | 0.119 | 0.129 | 0.124 | 0.110 |  | 0.126 | 0.120 | 0.115 |  |
| High school completion | | 5256 | 2844 | 846 | 827 | 739 | 0.11 | 1780 | 1713 | 1763 | 0.15 |
|  | | 0.603 | 0.593 | 0.621 | 0.605 | 0.623 |  | 0.601 | 0.592 | 0.617 |  |
| LGBT | | 1028 | 589 | 164 | 149 | 126 | 0.29 | 352 | 324 | 352 | 0.41 |
|  | | 0.118 | 0.123 | 0.120 | 0.109 | 0.106 |  | 0.119 | 0.112 | 0.123 |  |
| Chief complaint  Abdominal | | 1775 | 979 | 296 | 265 | 235 | 0.10 | 556 | 596 | 623 | 0.07 |
|  | | 0.204 | 0.204 | 0.217 | 0.194 | 0.198 |  | 0.188 | 0.206 | 0.218 |  |
| Cardiovascular | | 1020 | 544 | 176 | 167 | 133 |  | 339 | 329 | 352 |  |
|  | | 0.117 | 0.113 | 0.129 | 0.122 | 0.112 |  | 0.115 | 0.114 | 0.123 |  |
| Endocrine | | 107 | 61 | 16 | 12 | 18 |  | 32 | 42 | 33 |  |
|  | | 0.012 | 0.013 | 0.012 | 0.009 | 0.015 |  | 0.011 | 0.015 | 0.012 |  |
| General / other | | 572 | 288 | 88 | 106 | 87 |  | 282 | 290 | 269 |  |
|  | | 0.066 | 0.060 | 0.065 | 0.078 | 0.073 |  | 0.095 | 0.1 | 0.094 |  |
| GU / renal | | 509 | 302 | 69 | 71 | 67 |  | 163 | 157 | 189 |  |
|  | | 0.058 | 0.063 | 0.051 | 0.052 | 0.056 |  | 0.055 | 0.054 | 0.066 |  |
| Musculoskeletal | | 1388 | 763 | 210 | 212 | 203 |  | 505 | 446 | 437 |  |
|  | | 0.159 | 0.159 | 0.154 | 0.155 | 0.171 |  | 0.1706 | 0.154 | 0.153 |  |
| Stroke | | 30 | 18 | 2 | 5 | 5 |  | 9 | 13 | 8 |  |
|  | | 0.003 | 0.004 | 0.001 | 0.004 | 0.004 |  | 0.003 | 0.0045 | 0.003 |  |
| Neurologic  non-stroke | | 523 | 296 | 64 | 82 | 81 |  | 193 | 174 | 156 |  |
|  | | 0.060 | 0.062 | 0.047 | 0.060 | 0.068 |  | 0.065 | 0.06 | 0.055 |  |
| Oral / dental | | 129 | 69 | 21 | 17 | 22 |  | 47 | 40 | 42 |  |
|  | | 0.015 | 0.014 | 0.015 | 0.012 | 0.019 |  | 0.016 | 0.014 | 0.015 |  |
| Psychiatric | | 87 | 52 | 13 | 12 | 10 |  | 28 | 27 | 32 |  |
|  | | 0.010 | 0.011 | 0.010 | 0.009 | 0.008 |  | 0.010 | 0.009 | 0.011 |  |
| Respiratory | | 660 | 372 | 111 | 94 | 83 |  | 239 | 210 | 211 |  |
|  | | 0.076 | 0.078 | 0.081 | 0.069 | 0.070 |  | 0.081 | 0.072 | 0.074 |  |
| Skin | | 651 | 386 | 76 | 106 | 83 |  | 237 | 232 | 182 |  |
|  | | 0.075 | 0.080 | 0.056 | 0.078 | 0.070 |  | 0.080 | 0.080 | 0.063 |  |
| Substance use | | 196 | 92 | 46 | 33 | 25 |  | 54 | 66 | 76 |  |
|  | | 0.022 | 0.019 | 0.034 | 0.024 | 0.021 |  | 0.018 | 0.023 | 0.027 |  |
| Trauma | | 799 | 422 | 132 | 140 | 105 |  | 276 | 274 | 249 |  |
|  | | 0.092 | 0.088 | 0.097 | 0.102 | 0.088 |  | 0.093 | 0.0946 | 0.087 |  |
| Did not complete questionnaire | | 1689 | 940 | 268 | 238 | 243 | 0.22 | 581 | 585 | 523 | 0.17 |
|  | | 0.194 | 0.196 | 0.197 | 0.174 | 0.205 |  | 0.196 | 0.202 | 0.183 |  |
| Risk Category  Low | | 3510 | 1943 | 537 | 554 | 576 | 0.50 | 1164 | 1195 | 1151 | 0.88 |
|  | | 0.403 | 0.405 | 0.394 | 0.406 | 0.485 |  | 0.3932 | 0.4126 | 0.403 |  |
| Intermediate | | 4394 | 2388 | 695 | 697 | 614 |  | 1530 | 1433 | 1431 |  |
|  | | 0.504 | 0.498 | 0.510 | 0.510 | 0.517 |  | 0.5169 | 0.4948 | 0.501 |  |
| High | | 811 | 469 | 130 | 115 | 97 |  | 266 | 268 | 277 |  |
|  | | 0.0931 | 0.0977 | 0.0954 | 0.0842 | 0.0817 |  | 0.09 | 0.093 | 0.097 |  |
| Previously tested  for HIV | | 7049 | 3880 | 1105 | 1114 | 950 | 0.80 | 2398 | 2325 | 2326 | 0.57 |
|  | | 0.809 | 0.808 | 0.811 | 0.816 | 0.800 |  | 0.81 | 0.803 | 0.814 |  |
|  | |  |  |  |  |  |  |  |  |  |  |
| Observations | | 8,715 | 4,800 | 1,362 | 1,366 | 1,187 |  | 2960 | 2896 | 2859 |  |
| Number (%) unless otherwise noted | | | | | |  |  |  |  |  |  |
| *p* values: | Age calculated with Wald test with James' approximation | | | | | |  |  |  |  |  |
|  | Risk of infection tested using nonparametric test for trend across ordinal categories | | | | | | | | |  |  |
|  | All other variables tested with Pearson's chi2 | | | | | | | |  |  |  |
