# Supplementary material for: Cash incentives versus defaults for HIV testing: A randomized clinical trial
Source: PLoS One. 2018 Jul 6;13(7):e0199833. doi: 10.1371/journal.pone.0199833 (PMC6034801; doi:10.1371/journal.pone.0199833)
Supplement: S3 Table — (DOCX) [file pone.0199833.s004.docx]

**S3. Risk-specific results**

Table S3 presents the full results from the fully-interacted model from Table 2 in the manuscript. This is an ordinary least squares analysis of the association between treatments and likelihood of consenting to HIV test. Columns 1-4 replicate manuscript Table 2. Column 5 presents the association between intermediate-risk and high-risk patients and likelihood of consenting to an HIV test (low risk is the omitted category). Column 6 shows the results from a regression of HIV consent on incentives, risk categories, and the interaction between incentives and risk; Column 7 does the same for defaults and risk categories. Finally, Column 8 presents the fully-interacted model, which includes incentives, defaults, and risk categories, two-way interactions between each of these, and the three-way interaction between all three categories.

| **S3 Table. Test acceptance according to level of risk** | | | | | | | | | |  | |  | |
| --- | --- | --- | --- | --- | --- | --- | --- | --- | --- | --- | --- | --- | --- |
| VARIABLES | (1) | (2) | (3) | (4) | (5) | | (6) | | (7) | | (8) | |  |
| Incentives |  |  |  |  | |  | |  | |  | |  | |
| $1 | 0.00966 |  | 0.0123 | 0.0624** | |  | | -0.0136 | |  | | 0.0785* | |
|  | (0.016) |  | (0.016) | (0.025) | |  | | (0.024) | |  | | (0.041) | |
| $5 | 0.105*** |  | 0.106*** | 0.142*** | |  | | 0.0930*** | |  | | 0.129*** | |
|  | (0.017) |  | (0.016) | (0.029) | |  | | (0.025) | |  | | (0.044) | |
| $10 | 0.150*** |  | 0.147*** | 0.182*** | |  | | 0.134*** | |  | | 0.192*** | |
|  | (0.016) |  | (0.016) | (0.027) | |  | | (0.025) | |  | | (0.043) | |
| Defaults |  |  |  |  | |  | |  | |  | |  | |
| Active choice |  | 0.115*** | 0.117*** | 0.133*** | |  | |  | | 0.135*** | | 0.160*** | |
|  |  | (0.013) | (0.013) | (0.018) | |  | |  | | (0.020) | | (0.027) | |
| Opt out |  | 0.239*** | 0.239*** | 0.279*** | |  | |  | | 0.252*** | | 0.312*** | |
|  |  | (0.013) | (0.013) | (0.017) | |  | |  | | (0.020) | | (0.027) | |
| Incentives x Defaults |  |  |  |  | |  | |  | |  | |  | |
| $1 x Active choice | |  |  | -0.0708** | |  | |  | |  | | -0.135** | |
|  |  |  |  | (0.035) | |  | |  | |  | | (0.057) | |
| $1 x Opt out |  |  |  | -0.0827** | |  | |  | |  | | -0.134** | |
|  |  |  |  | (0.036) | |  | |  | |  | | (0.059) | |
| $5 x Active choice | |  |  | -0.0229 | |  | |  | |  | | 0.0188 | |
|  |  |  |  | (0.037) | |  | |  | |  | | (0.057) | |
| $5 x Opt out |  |  |  | -0.0857** | |  | |  | |  | | -0.109* | |
|  |  |  |  | (0.040) | |  | |  | |  | | (0.061) | |
| $10 x Active choice | |  |  | -0.00592 | |  | |  | |  | | -0.0230 | |
|  |  |  |  | (0.038) | |  | |  | |  | | (0.062) | |
| $10 x Opt out |  |  |  | -0.0952*** | |  | |  | |  | | -0.143** | |
|  |  |  |  | (0.036) | |  | |  | |  | | (0.061) | |
| Risk of infection |  |  |  |  | |  | |  | |  | |  | |
| Intermediate risk | |  |  |  | | 0.0713*** | | 0.0590*** | | 0.0806*** | | 0.0856*** | |
|  |  |  |  |  | | (0.011) | | (0.015) | | (0.019) | | (0.025) | |
| High risk |  |  |  |  | | 0.0905*** | | 0.0773*** | | 0.160*** | | 0.158*** | |
|  |  |  |  |  | | (0.019) | | (0.026) | | (0.034) | | (0.045) | |
| Incentives x Risk |  |  |  |  | |  | |  | |  | |  | |
| $1 x intermediate risk | |  |  |  | |  | | 0.0410 | |  | | -0.0197 | |
|  |  |  |  |  | |  | | (0.032) | |  | | (0.054) | |
| $1 x high risk |  |  |  |  | |  | | 0.0186 | |  | | -0.0422 | |
|  |  |  |  |  | |  | | (0.053) | |  | | (0.099) | |
| $5 x intermediate risk | |  |  |  | |  | | 0.0188 | |  | | 0.0246 | |
|  |  |  |  |  | |  | | (0.033) | |  | | (0.059) | |
| $5 x high risk |  |  |  |  | |  | | 0.0287 | |  | | 0.0601 | |
|  |  |  |  |  | |  | | (0.056) | |  | | (0.100) | |
| $10 x intermediate risk | | |  |  | |  | | 0.0165 | |  | | -0.0251 | |
|  |  |  |  |  | |  | | (0.031) | |  | | (0.058) | |
| $10 x high risk |  |  |  |  | |  | | 0.0824 | |  | | 0.0523 | |
|  |  |  |  |  | |  | | (0.056) | |  | | (0.095) | |
| Defaults x Risk |  |  |  |  | |  | |  | |  | |  | |
| Active choice x intermediate risk | | | |  | |  | |  | | -0.0219 | | -0.0330 | |
|  |  |  |  |  | |  | |  | | (0.027) | | (0.036) | |
| Active choice x high risk | | |  |  | |  | |  | | -0.0792* | | -0.0817 | |
|  |  |  |  |  | |  | |  | | (0.048) | | (0.064) | |
| Opt out x intermediate risk | | |  |  | |  | |  | | 0.000874 | | -0.0320 | |
|  |  |  |  |  | |  | |  | | (0.027) | | (0.036) | |
| Opt out x high risk | |  |  |  | |  | |  | | -0.133*** | | -0.154** | |
|  |  |  |  |  | |  | |  | | (0.047) | | (0.064) | |
| Incentives x Defaults x Risk | | |  |  | |  | |  | |  | |  | |
| $1 x |  |  |  |  | |  | |  | |  | |  | |
| Active choice x Intermediate risk | | | |  | |  | |  | |  | | 0.0934 | |
|  |  |  |  |  | |  | |  | |  | | (0.076) | |
| Active choice x High risk | | |  |  | |  | |  | |  | | 0.116 | |
|  |  |  |  |  | |  | |  | |  | | (0.133) | |
| Opt out x Intermediate risk | | |  |  | |  | |  | |  | | 0.0926 | |
|  |  |  |  |  | |  | |  | |  | | (0.076) | |
| Opt out x Intermediate risk | | |  |  | |  | |  | |  | | 0.0462 | |
|  |  |  |  |  | |  | |  | |  | | (0.141) | |
|  |  |  |  |  | |  | |  | |  | |  | |
| $5 x |  |  |  |  | |  | |  | |  | |  | |
| Active choice x Intermediate risk | | | |  | |  | |  | |  | | -0.0669 | |
|  |  |  |  |  | |  | |  | |  | | (0.079) | |
| Active choice x High risk | | |  |  | |  | |  | |  | | -0.148 | |
|  |  |  |  |  | |  | |  | |  | | (0.137) | |
| Opt out x Intermediate risk | | |  |  | |  | |  | |  | | 0.0277 | |
|  |  |  |  |  | |  | |  | |  | | (0.078) | |
| Opt out x Intermediate risk | | |  |  | |  | |  | |  | | 0.0351 | |
|  |  |  |  |  | |  | |  | |  | | (0.134) | |
| $10 x |  |  |  |  | |  | |  | |  | |  | |
| Active choice x Intermediate risk | | | |  | |  | |  | |  | | 0.0237 | |
|  |  |  |  |  | |  | |  | |  | | (0.081) | |
| Active choice x High risk | | |  |  | |  | |  | |  | | 0.0289 | |
|  |  |  |  |  | |  | |  | |  | | (0.135) | |
| Opt out x Intermediate risk | | |  |  | |  | |  | |  | | 0.0746 | |
|  |  |  |  |  | |  | |  | |  | | (0.079) | |
| Opt out x Intermediate risk | | |  |  | |  | |  | |  | | 0.0633 | |
|  |  |  |  |  | |  | |  | |  | | (0.126) | |
| Constant | 0.516*** | 0.437*** | 0.399*** | 0.380*** | | 0.510*** | | 0.479*** | | 0.381*** | | 0.320*** | |
|  | (0.008) | (0.010) | (0.011) | (0.013) | | (0.009) | | (0.012) | | (0.015) | | (0.019) | |
|  |  |  |  |  | |  | |  | |  | |  | |
| Observations | 8,715 | 8,715 | 8,715 | 8,715 | | 8,715 | | 8,715 | | 8,715 | | 8,715 | |
| Each column shows percentage point difference in HIV test acceptance estimated from an ordinary least squares regression. Dependent variable = acceptance of HIV test. | | | | | | | | | | | | | |
| Omitted categories for incentives, defaults, and risk groups are No incentive, opt-in testing, and low risk, respectively. | | | | | | | | | | | | | |
| Standard errors are clustered at day-zone level. | | | | | | | | | | | | | |
| *** p<0.01, ** p<0.05, * p<0.1 | | | | | | | | | | | | | |
