# Supplementary material for: Cash incentives versus defaults for HIV testing: A randomized clinical trial
Source: PLoS One. 2018 Jul 6;13(7):e0199833. doi: 10.1371/journal.pone.0199833 (PMC6034801; doi:10.1371/journal.pone.0199833)
Supplement: S4 Table — (DOCX) [file pone.0199833.s005.docx]

**S4 Table. Alternate models**

To address concerns that the linear model used to estimate effect sizes may have produced different results than a nonlinear binary model would have, we present the treatment effects below using three alternate approaches for a representative specification. Due to the fact that the test acceptance rate is in the middle part of the distribution, results are quite similar if instead using a nonlinear model such as logistic regression. Table S4 reports the results from a specification that includes incentives, defaults, and risk category (omitted category no incentive, opt-in, and low risk, respectively) using a linear probability model (ordinary least squares), logit with estimated marginal effects, and logit with odds ratios. Columns 1 and 2 are virtually identical, supporting our use of a linear probability model.

| **S4 Table. Treatment effects: OLS and logistic** | | |  |
| --- | --- | --- | --- |
|  | (1) | (2) | (3) |
| VARIABLES | OLS | Logit  marginal effects | Logit  odds ratios |
| Incentives |  |  |  |
| $1 | 0.0116 | 0.012 | 1.05 |
|  | (0.016) | (0.016) | (0.070) |
| $5 | 0.106*** | 0.106*** | 1.58*** |
|  | (0.016) | -0.016 | (0.113) |
| $10 | 0.147*** | 0.147*** | 1.90*** |
|  | (0.016) | -0.016 | (0.137) |
| Defaults |  |  |  |
| Active choice | 0.118*** | 0.118*** | 1.62*** |
|  | (0.013) | (0.013) | (0.085) |
| Opt out | 0.239*** | 0.239*** | 2.75*** |
|  | (0.013) | (0.013) | (0.155) |
| Risk of infection | |  |  |
| Intermediate risk | 0.0728*** | 0.073*** | 1.365*** |
|  | (0.011) | (0.106) | (0.062) |
| High risk | 0.0924*** | 0.092*** | 1.485*** |
|  | (0.019) | (0.019) | (0.123) |
| Dependent variable = acceptance of HIV test. | | | |
| Omitted categories for incentives, defaults, and risk groups are no incentive, opt-in testing, and low risk, respectively. | | | |
| Standard errors are clustered at day-zone level. | | | |
| *** p<0.01, ** p<0.05, * p<0.1 | | |  |
