# Supplementary material for: Cash incentives versus defaults for HIV testing: A randomized clinical trial
Source: PLoS One. 2018 Jul 6;13(7):e0199833. doi: 10.1371/journal.pone.0199833 (PMC6034801; doi:10.1371/journal.pone.0199833)
Supplement: S5 Table — (DOCX) [file pone.0199833.s006.docx]

**S5 Table. Sensitivity Analysis**

Because of the retrospective consent design with slightly differential study consent rates across incentive arms, we tested sensitivity to bounding estimated treatment effects under alternative counterfactual study consent assumptions. Patients in the $10 treatment assignment were significantly more likely to agree to participate in the study than those in the no incentive group; these groups likewise had significantly different HIV consent rates. Thus, we estimated the lower bound on the HIV consent percentage among patients assigned to the $10 incentive, denoted “Sample $10 assignment.” This group was created from the $10 incentive group, adjusted as if they enrolled in the study at the same proportion as those in the no incentive group (as if enrollment were 82.6% instead of the observed 88.3%, S5 Table), with the assumption that the patients removed from this group all consented to the test.

| **S5 Table.** Percentage consenting to study inclusion according to monetary treatment  assignment | | | | | |
| --- | --- | --- | --- | --- | --- |
|  | Incentive Treatment Assignment | | | | |
|  | **None** | **$1** | **$5** | **$10** | **All** |
| **Declined** | 1025 | 258 | 221 | 157 | 1661 |
| **Approached** | 5904 | 1624 | 1592 | 1343 | 10463 |
| **Study consent** | 82.6% | 84.1% | 86.1% | 88.3% | 84.1% |
