# Supplementary material for: Cash incentives versus defaults for HIV testing: A randomized clinical trial
Source: PLoS One. 2018 Jul 6;13(7):e0199833. doi: 10.1371/journal.pone.0199833 (PMC6034801; doi:10.1371/journal.pone.0199833)
Supplement: S6 Table — (DOCX) [file pone.0199833.s007.docx]

**S6 Table. Sensitivity Analysis**

Because of the retrospective consent design with slightly differential study consent rates across incentive arms, we tested sensitivity to bounding estimated treatment effects under alternative counterfactual study consent assumptions. Patients in the $10 treatment assignment were significantly more likely to agree to participate in the study than those in the no incentive group; these groups likewise had significantly different HIV consent rates. Thus, we estimated the lower bound on the HIV consent percentage among patients assigned to the $10 incentive, denoted “Sample $10 assignment.” This group was created from the $10 incentive group, adjusted as if they enrolled in the study at the same proportion as those in the no incentive group (as if enrollment were 82.6% instead of the observed 88.3%, S5 Table), with the assumption that the patients removed from this group all consented to the test.

Eliminating 76 patients who we assume to have accepted the HIV test yields a $10 incentive group with 714 out of 1111 (64.3%) patients accepting HIV testing, with a difference between the hypothetical $10 treatment and the no incentive group of 12.7 percentage points (Chi2 p < 0.001). Even with this extreme assumption that all of the additional patients in the $10 treatment arm accepted the test, this simulated effect is quite similar to our actual estimated effect size, indicating that results are highly robust to the minor differences in study consent rates across arms.

| **S6 Table.** HIV consent according to monetary incentive | | | | |  |  |
| --- | --- | --- | --- | --- | --- | --- |
|  | Incentive Treatment Assignment | | | | | |
|  | **None** | **$1** | **$5** | **$10** | **All** | **Sample $10 assignment*** |
| **HIV test acceptance** | 51.6% | 52.6% | 62.1% | 66.6% | 55.4% | 64.3% |
| **Consent to test** | 2477 | 716 | 848 | 790 | 4831 | 714 |
| **Sample size** | 4800 | 1362 | 1366 | 1187 | 8715 | 1111 |
| * Remove 76 subjects from the $10 cohort, all of whom consented to test | | | | |  |  |
